# Estrogen-Induced Extracellular Calcium Influx Promotes Endometrial Cancer Progress by Regulating Lysosomal Activity and Mitochondrial ROS

**DOI:** 10.3389/fmed.2022.835700

**Published:** 2022-02-10

**Authors:** Boqiang Shen, Juan Hao, Yanying Lin, Xingchen Li, Xiao Yang, Ting Huang, Jiaqi Wang, Yuanyuan Jia, Jingyi Zhou, Jianliu Wang

**Affiliations:** ^1^Department of Obstetrics and Gynecology, Peking University People's Hospital, Beijing, China; ^2^Center of Reproductive Medicine, Fujian Maternity and Child Health Hospital, Affiliated Hospital of Fujian Medical University, Fuzhou, China

**Keywords:** endometrial cancer (EC), estrogen, serum calcium, calcium homeostasis, prognosis (carcinoma)

## Abstract

**Objective:**

Calcium is present in serum mainly in filterable and bound forms, and Ca^2+^ is a major key to modulate signaling pathways that control oncogenesis and oncochannels associated with several types of cancer. However, the biological significance of serum calcium and its related mechanism with estrogen in endometrial cancer (EC) still remains elusive. This study aims to ascertain the relationship between serum calcium and clinicopathology in EC.

**Methods:**

Retrospective assessment of a total of 502 patients diagnosed with EC after surgery in Peking University People's Hospital from 2010 to 2018. Preoperative serum ionized calcium and the albumin corrected calcium was calculated in quartiles for various postoperative clinicopathological characteristics, logistic regression adjusted for potential confounders. Intracellular calcium homeostasis change induced by estrogen was detected by confocal analysis. Downstream pathways were analyzed by transcriptome and proteomics. Mitochondrial Ca^2+^ and ROS (reactive oxygen species) level was detected by confocal and flow cytometry. Lysosomal morphological and membrane changes were verified by confocal or Western blot assays.

**Results:**

High level of albumin-corrected serum calcium was significantly correlated with EC clinicopathological characteristics progression include lymph vascular space invasion, lymph nodes metastasis, myometrial invasion, and cervical invasion. Calcium homeostasis regulated by estrogen in EC cells derived from extracellular calcium influx but not the release of the endoplasmic reticulum. Proteomic and bioinformatic analysis revealed the calcium influx might be involved in the regulation of autophagy and mitochondrial-related pathways. Mechanistic investigation demonstrated that calcium influx acted on the function of mitochondrial ROS and lysosomal activity.

**Conclusion:**

Our findings revealed that serum calcium level was significantly related to poor outcomes. The extracellular calcium influx induced by estrogen was targeted to mitochondrial ROS and lysosome activity, which should be oriented to improve EC therapeutic strategies.

## Introduction

Endometrial cancer (EC) is a common female reproductive system tumor originating from the endometrium epithelium. Research shows that in 2021, the number of new cases of endometrial cancer in the United States is the fourth in female malignant tumors and the sixth in death (1). Cancer survival has improved since the mid-1970s for all of the most common cancers except uterine cervix and uterine corpus, mainly reflecting the absence of significant treatment advances for these cancers (1). EC lags behind nearly every primary cancer type in terms of developing targeted therapy. To date, predictive biomarkers have not been identified (2). Therefore, it is urgent to understand better the molecular mechanisms that govern the oncogenesis and progression of EC.

The prognosis of low-grade endometrioid histology is generally good, while non-endometrioid tumors and high-grade endometrioid disease carry a markedly poorer prognosis. Driven by initiatives of ProMisE and Trans PORTEC, the design and validation of combined molecular classifiers is ongoing (3, 4). Compared to the tissue-based biomarkers, blood-based biomarkers represent less invasive clinical tools to predict prognosis and plan patient treatment, like Ca-125, which has prognostic value and identifies advanced disease and lymph node metastasis (5, 6). Although the estrogen receptor (ER) expression on prognosis and the estrogen on the risk of acquiring disease have been extensively researched, we have previously demonstrated that non-nuclear ER to account for estrogen action in the plasma membrane and calcium mobilization (7), few studies have evaluated the importance of other blood-based biomarkers like the calcium levels. Calcium is present in serum mainly in filterable and bound forms, and Ca^2+^ is significant to modulating signaling pathways that control oncogenesis and cancer progression (8). Based on the previous work, we have found that there is a significant positive correlation between serum calcium and high-density lipoprotein, low-density lipoprotein, and total cholesterol in patients with endometrial cancer (9), and increased serum calcium is involved in the regulation of lymph node metastasis in endometrial cancer (10). In this study, we analyze the differences in serum calcium to identify links between serum calcium and the clinicopathological characteristics of endometrial cancer. The study aimed to evaluate the prognostic value of serum calcium levels in endometrial cancer patients and the mechanisms involved in regulating intracellular calcium levels and organelle calcium homeostasis if elevated serum calcium occurs.

## Materials and Methods

### Patients

All data were collected from medical records of all patients who were histopathological and clinically diagnosed with EC between January 2010 to December 2018 in Peking University People's hospital. Patient characteristics and various clinicopathologic data were obtained and reviewed retrospectively. EC patients with a parathyroid disease or other that affect serum calcium levels were excluded, as well as those whose pathology was accompanied by other cancers or recurrent disease and those whose preoperative venous blood biochemistry indicators were missing. This study was approved by the Medical Ethics Committee of Peking University People's Hospital.

### Serum Calcium Analysis

An accurate estimate of the calcium that is biologically active depends upon the concentration of serum albumin, as only the unbound (“free” or “ionized”) fraction of serum calcium is active. Calcium and albumin were measured by an automatic biochemical analyzer (Beckman AU580028, Department of Laboratory Medicine, Peking University People's Hospital). The biologically active fraction of serum calcium (ionized calcium) was calculated by the following formula: Corrected serum calcium (mmol/L) = serum calcium (mmol/L) +0.2^*^[40- Serum albumin (g/L)] (11). Select appropriate serum calcium cut-off points for postoperative clinicopathological single-factor analysis, and clarify the predictive value of four-category preoperative serum ionized calcium and corrected serum calcium level for various postoperative clinicopathological characteristics.

### Statistical Analysis

Continuous variables of descriptive data are represented by mean (MEAN) ± standard deviation (SD) or median (interquartile range, IQR), and categorical variables are represented as number and percentage (%). Statistical differences between paired experimental groups were examined by two-tailed independent sample student's t-test. One-way analysis of variance (ANOVA) with Bonferroni post hoc testing was examined for the case of more than two groups. Chi-square test (Chi-square test) or Fisher's exact tests were examined to compare the differences of categorical variables. Mann–Whitney U test was used to analyze the difference between the means and proportions of the two groups. Statistical differences were considered as significant for *p* < 0.05. All statistical analysis was done using SPSS 22.0 software or Prism 8 software (GraphPad).

### Proteomics/Transcriptomics and Bioinformatics Analysis

Total RNA extraction-mRNA isolation-library building reagents-quantification-library recovery-bridge amplification-on-machine sequencing; the analysis process is output data-data removal-transcriptome splicing-SSR analysis and SNP analysis-gene function annotation- Gene expression differential analysis-differential gene expression pattern clustering-differential gene enrichment analysis. Significant DEGs were annotated using the Kyoto Encyclopedia of Genes and Genomes (KEGG). A gene ontology (GO) analysis was performed using standalone Blast2GO v3.2 with the same E-value cut-off. This software assigned GO terms to each DEG to allow their putative functions to be predicted in terms of molecular functions (MFs), biological processes (BPs), and cellular components (CCs).

### Cell Culture

The experiment introduced five human endometrial cancer cell lines (Ishikawa, RL-952, HEC-1A, KLE, AN3CA), which were cryopreserved in the Gynecological Oncology Laboratory of Peking University People's Hospital. The culture condition is a constant temperature cell incubator at 37°C, 5% CO2, and saturated humidity. The five cell lines are all adherent cells. Cells were subcultured every 2–3 days and used for cell-based experiments.

### Calcium Influx Detection With Fluo 4-AM

The concentration of intracellular Ca^2+^ was determined using a cell-permeable fluorescent calcium indicator, Fluo 4-AM. Cells were treated with DMEM-F12 and 10% bovine serum for 24 h and washed three times with D-Hanks balanced salt solution without Ca^2+^ (Ca^2+^-free HBSS). Subsequently, cells were loaded with 2 μmol/l Fluo 4-AM for 30 min at 37 °C in the dark, then washed three times with Ca^2+^-free HBSS to remove the extracellular Fluo 4-AM. The solution was replaced with HBSS/D-hanks/D-hanks with a specific calcium chloride solution before testing. Imaging was performed using the Leica SP8 Confocal Inverted Microscopy (Leica, Mannheim, Germany) and analyzed with Image-Pro Plus 6.0. The first 60s were recorded as the base calcium level. We used the measured average fluorescence intensity of each cell in the field (F), normalized to the non-specific background fluorescence (F0) to obtain the fluorescence intensity (F/F0).

### Detection of Intracellular Oxidative Stress by Flow Cytometry

Intracellular oxidative stress of calcium influx induced by E2-BSA treatment was determined by flow cytometry using 2′,7′-dichlorofluorescein-diacetate (DCFH-DA). Cells were seeded on 6-well plates at (1.25 × 10^5^) cells per well and cultured overnight. After that, cells were incubated with 50 μM DCFH-DA for 20 min in an incubator following an appropriate wash with phosphate-buffered saline (PBS) as protocol, then treated with 100 nm E2-BSA for 30mins, 0.1% DMSO alone as a negative control and 100 μM ROS-UP as a positive control. Then centrifuge and resuspend the cells by digestion. The fluorescence was determined by Flowcytometer FACSCalibur (BD Biosciences, NJ, USA), with excitation at 480 nm and emission at 525 nm.

### Cathepsin Activity Fluorometric Assay

The enzymatic activities of and cathepsin B (CTSB) in Ishikawa cells and HEC-1A cells were tested using the CTSB activity fluorometric assay kit (No. ab65300, Abcam, USA). Briefly, cells were collected (1 × 10^6^) by centrifugation. Lysed cells in 50 μl of cell lysis buffer and incubated cells on ice for 10 min. Centrifuge at 20,000 g for 5 min, and then transferred the supernatant to a new tube. Added 50 μl of cell lysate to the opaque black 96-well plate. Then 50 μl of reaction buffer and 2 μl of the 10 mM substrate Ac-RR-AFC were added to each sample. 2 μl of inhibitor for negative control. Enspire multifunctional microplate reader was used with 400- nm excitation and 505- nm emission filter to analyze fluorescence intensity for CTSB enzymatic activities after incubating at 37°C for 2 h in the dark.

### Lyso-Tracker Red Staining

Lysosomal staining was performed using Lyso-Tracker Red (LTR) purchased from Invitrogen (Massachusetts, USA). After 6 h of treatment with E2-BSA in a 20-mm glass-bottom confocal dish, cells (1.0 × 10^6^ cells/ml) were incubated with LysoTracker Red at 5 nmol/L for 30 min at 37°C as the user manual and washed three times with phosphate-buffered saline (PBS). The cells were then inspected and photographed with a TCS SP8 STED confocal microscope (Leica, Germany).

### Acridine Orange Staining

After the designated treatments in a 20-mm glass-bottom dish, cells (1.0 × 106 cells/ml) were incubated with a medium containing 5 μg/ml acridine orange (AO) for 15 min at 37°C and rinsed with PBS. Then the cells were imaged under the confocal microscope with the excitation wavelength set at 488 and 555nm; two separate emission bands (505–550 and 600–650 nm) were obtained separately.

### Western Blot Analysis

After protein quantification with a bicinchoninic acid (BCA) protein assay kit (Thermo Fisher Scientific), a total of 30 μg of denatured proteins per line underwent electrophoresis. Then, the proteins were transferred to polyvinylidene fluoride (PVDF) membranes and blocked by 5% (w/v) milk (non-fat milk in Tris-buffered saline with Tween 20 [TBST]). Membranes were incubated in primary antibodies against LAMP1, LAMP2, Cathespin B, TFEB, β-Actin, GAPDH at 4°C overnight. The following day, after washing with TBST three times, membranes were incubated in peroxidase-conjugated secondary antibodies for 1 h at room temperature. After washing with TBST for another 3 times, the detection of protein bands was performed with an enhanced chemiluminescence system (Bio-Rad, USA).

## Results

### Patients and Clinicopathological Characteristics

**Figure 1:**
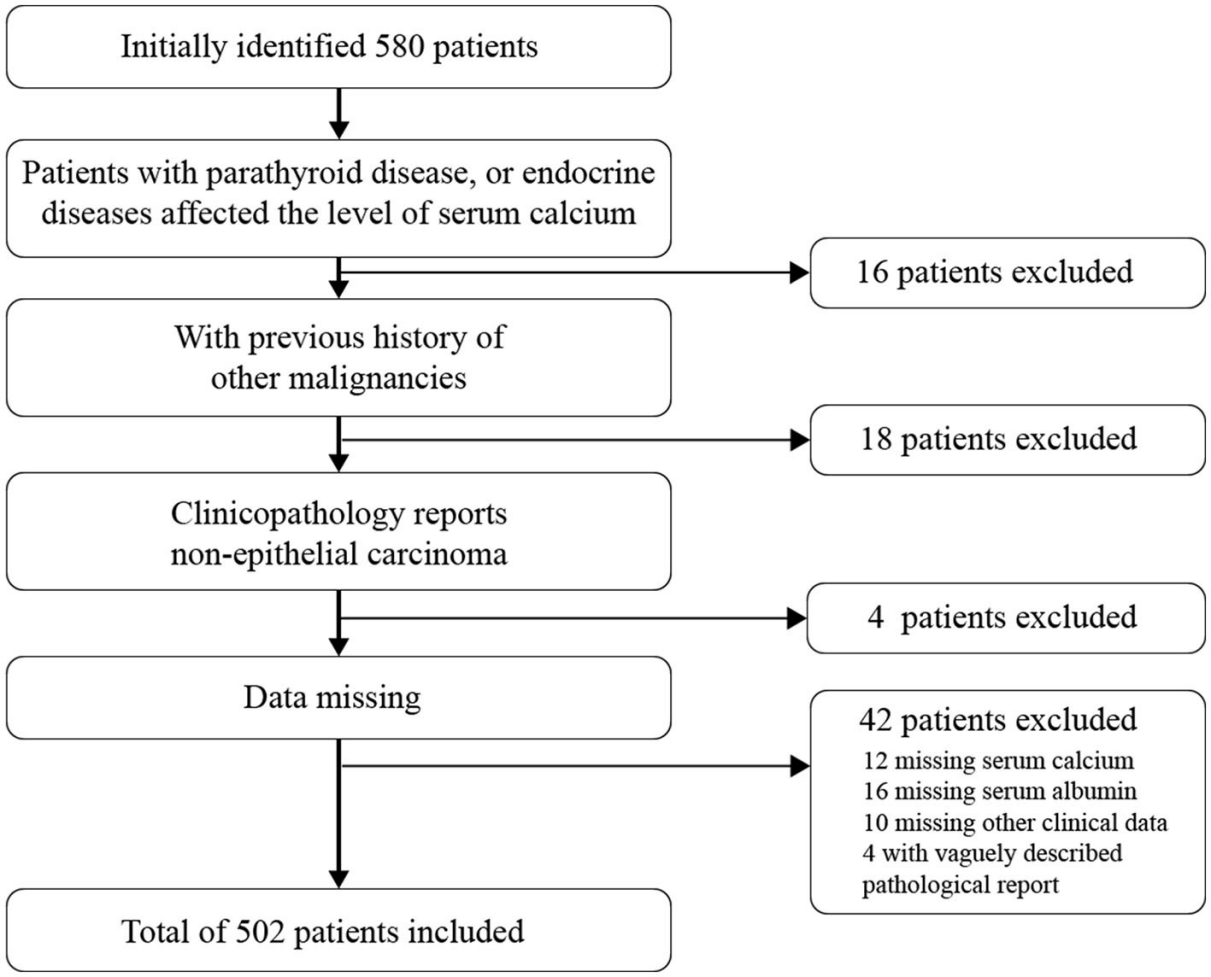
Patient selection for EC patients.

We identified 580 cases diagnosed with EC after bringing in the exclusion criterion (Figure 1) due to irrelevant malignant history or missing data (*N* = 78). During 2010–2018, data remained for 502 participants (86.56%). Patients' demographic and clinical characteristics are described in Table 1 stratification by quartiles. The average age of the patients was 55.84 ± 9.57 years old, and the average body mass index (kg/m^2^) was 26.3 ± 4.61. A total of 320 (63.75%) patients were postmenopausal women at diagnosis. 57 cases (11.35%) had lymph node metastasis, 87 cases (17.33%) had lymph vascular space invasion (LVSI), 164 cases (32.67%) had myometrial invasion, and 101 cases (20.12%) had a cervical invasion. Among the biochemical indicators, the serum calcium concentration of the enrolled patients was 2.01 ± 0.33 mmol/L, and albumin was 43.42 ± 4.4 g/L. After the data were corrected by formula with albumin, the calcium ion was divided into quartiles for four classifications, and the Q1-Q4 levels were obtained and grouped. The results in quartile positively correlated with the increase of ionized calcium and menopausal status (*P* = 0.014), age (*P* = 0.004), FIGO staging (*P* = 0.012), lymph node metastasis (*P* = 0.001), LVSI (*P* = 0.011), myometrial invasion (*P* = 0.002), and cervical invasion (*P* = 0.017).

**Table 1:** Demographic characteristics for EC patients according to quartiles of serum calcium and corrected serum calcium.

	**Total** ***N*** **= 502**	**Quartiles of serum calcium (mmol/L)**					**Quartiles of corrected calcium (mmol/L)**			
		**Quartile 1**	**Quartile 2**	**Quartile 3**	**Quartile 4**	**Trend p**	**Quartile 1**	**Quartile 2**	**Quartile 3**	**Quartile 4**	**Trend p**
		**(≤2.20;** ***N =*** **130)**	**(2.21–2.30;** ***N =*** **128)**	**(2.31–2.38;** ***N =*** **122)**	**(>2.39;** ***N =*** **122)**		**(≤1.81;** ***N =*** **126)**	**(1.82-1.95;** ***N =*** **125)**	**(1.96–2.12;** ***N =*** **127)**	**(>2.12;** ***N =*** **142)**	
Age (yr)	55.84 ± 9.57	55.09 ± 9.43	55.27 ± 10.02	57.07 ± 9.45	55.98 ± 9.34	0.241	55.09 ± 9.43	55.5 ± 9.25	56.43 ± 10.11	57.37 ± 9.59	0.004
<40	26 (5.18)	7 (5.38)	8 (6.25)	5 (4.1)	6 (4.92)		9 (7.14)	6 (4.8)	7 (5.51)	4 (3.23)	
40-50	114 (22.71)	38 (29.23)	32 (25)	21 (17.21)	23 (18.85)		34 (26.98)	31 (24.8)	26 (20.47)	23 (18.55)	
50-60	208 (41.43)	46 (35.38)	53 (41.41)	53 (43.44)	56 (45.9)		55 (43.65)	55 (44)	55 (43.31)	43 (34.68)	
60-70	125 (24.9)	33 (25.38)	27 (21.09)	34 (27.87)	31 (25.41)		24 (19.05)	27 (21.6)	27 (21.26)	47 (37.9)	
>70	29 (5.78)	6 (4.62)	8 (6.25)	9 (7.38)	6 (4.92)		4 (3.17)	6 (4.8)	12 (9.45)	7 (5.65)	
BMI (kg/m2)	26.3 ± 4.61	26.1 ± 4.96	26.22 ± 4.08	26.88 ± 5.24	26.01 ± 4.06	0.830	25.9 ± 4.51	26.22 ± 4.08	26.88 ± 5.24	26.41 ± 4.78	0.410
<24	152 (30.28)	41 (31.54)	33 (25.78)	38 (31.15)	40 (32.79)		46 (36.51)	32 (25.6)	38 (29.92)	36 (29.03)	
24-28	196 (39.04)	53 (40.77)	52 (40.63)	42 (34.43)	49 (40.16)		48 (38.1)	52 (41.6)	49 (38.58)	47 (37.9)	
>28	154 (30.68)	36 (27.69)	43 (33.59)	42 (34.43)	33 (27.05)		32 (25.4)	41 (32.8)	40 (31.5)	41 (33.06)	
Menopausal status						0.014					0.236
No	182 (36.25)	55 (42.31)	53 (41.41)	37 (30.33)	37 (30.33)		50 (39.68)	46 (36.8)	46 (36.22)	40 (32.26)	
Yes	320 (63.75)	75 (57.69)	75 (58.59)	85 (69.67)	85 (69.67)		76 (60.32)	79 (63.2)	81 (63.78)	84 (67.74)	
Grade						0.320					0.062
I	165 (32.87)	41 (31.54)	42 (32.81)	49 (40.16)	33 (27.05)		38 (30.16)	48 (38.4)	46 (36.22)	33 (26.61)	
II	218 (43.43)	58 (44.62)	61 (47.66)	49 (40.16)	50 (40.98)		65 (51.59)	53 (42.4)	46 (36.22)	54 (43.55)	
III	119 (23.71)	31 (23.85)	25 (19.53)	24 (19.67)	39 (31.97)		23 (18.25)	24 (19.2)	35 (27.56)	37 (29.84)	
FIGO 2009						0.073					0.012
I	398 (79.28)	105 (80.77)	104 (81.25)	100 (81.97)	89 (72.95)		104 (82.54)	102 (81.6)	106 (83.46)	86 (69.35)	
II	25 (4.98)	7 (5.38)	4 (3.13)	8 (6.56)	6 (4.92)		7 (5.56)	3 (2.4)	5 (3.94)	10 (8.06)	
III	64 (12.75)	17 (13.08)	15 (11.72)	14 (11.48)	18 (14.75)		14 (11.11)	17 (13.6)	13 (10.24)	20 (16.13)	
IV	15 (2.99)	1 (0.77)	5 (3.91)	0 (0)	9 (7.38)		1 (0.79)	3 (2.4)	3 (2.36)	8 (6.45)	
Histologic subtype						0.296					0.126
EEC	432 (86.06)	112 (86.15)	114 (89.06)	106 (86.89)	100 (81.97)		115 (91.27)	106 (84.8)	106 (83.46)	105 (84.68)	
NEEC	70 (13.94)	18 (13.85)	14 (10.94)	16 (13.11)	22 (18.03)		11 (8.73)	19 (15.2)	21 (16.54)	19 (15.32)	
LNM						0.303					0.001
No	445 (88.65)	116 (89.23)	114 (89.06)	113 (92.62)	102 (83.61)		118 (93.65)	113 (90.4)	115 (90.55)	99 (79.84)	
Yes	57 (11.35)	14 (10.77)	14 (10.94)	9 (7.38)	20 (16.39)		8 (6.35)	12 (9.6)	12 (9.45)	25 (20.16)	
LVSI						0.112					0.011
No	415 (82.67)	110 (84.62)	107 (83.59)	106 (86.89)	92 (75.41)		115 (91.27)	103 (82.4)	102 (80.31)	95 (76.61)	
Yes	87 (17.33)	20 (15.38)	21 (16.41)	16 (13.11)	30 (24.59)		11 (8.73)	22 (17.6)	25 (19.69)	29 (23.39)	
Myometrial invasion						0.993					0.002
No	338 (67.33)	83 (63.85)	90 (70.87)	83 (69.75)	78 (63.93)		90 (71.43)	94 (75.2)	86 (67.72)	68 (54.84)	
Yes	164 (32.67)	47 (36.15)	37 (29.13)	36 (30.25)	44 (36.07)		36 (28.57)	31 (24.8)	41 (32.28)	56 (45.16)	
Cervical invasion						0.123					0.017
No	401 (79.88)	106 (81.54)	104 (81.89)	99 (83.19)	89 (72.95)		104 (82.54)	104 (83.2)	107 (84.25)	86 (69.35)	
Yes	101 (20.12)	24 (18.46)	23 (18.11)	20 (16.81)	33 (27.05)		22 (17.46)	21 (16.8)	20 (15.75)	38 (30.65)	
Peritoneal cytology						0.079					0.064
No	466 (92.83)	124 (95.38)	117 (92.13)	111 (93.28)	110 (90.16)		122 (96.83)	115 (92)	115 (90.55)	114 (91.94)	
Yes	36 (7.17)	6 (4.62)	10 (7.87)	8 (6.72)	12 (9.84)		4 (3.17)	10 (8)	12 (9.45)	10 (8.06)	
Albumin (g/L)	43.42 ± 4.4	41.05 ± 5.45	43.58 ± 3.62	44.46 ± 3.19	44.78 ± 3.91	<0.0001	47.41 ± 1.91	43.58 ± 3.62	43.34 ± 1.68	37.76 ± 4.31	<0.0001
CA125 (U/ml)	34.04 ± 48.06	30.36 ± 38.98	32.36 ± 50.68	33.04 ± 44.98	40.41 ± 56.19	0.163	26.67 ± 24.85	33.19 ± 41.1	33.9 ± 50.36	42.24 ± 65.46	0.697
CA199 (U/ml)	27.67 ± 46.40	26.91 ± 64.89	25.34 ± 38.1	28.32 ± 29.88	29.64 ± 48.77	0.640	24.29 ± 29.1	24.38 ± 29.76	27.31 ± 41.17	36.6 ± 77.57	0.102
Corrected calcium (mmol/L)	2.01 ± 0.33	2.02 ± 0.42	1.97 ± 0.29	1.99 ± 0.25	2.07 ± 0.34	<0.0001	–	–	–	–	

*The quantitative variables were described as number (%), mean ± standard deviation (SD), or median (interquartile range, IQR). Categorical variables were presented as proportions. BMI, body mass index; FIGO, International Federation of Gynecology and Obstetrics; EEC, endometrioid endometrial carcinoma NEEC, non-endometrioid endometrial carcinoma; LNM, lymph node metastasis; LVSI, Lymph vascular space invasion. Linear trend across quartiles of serum calcium was tested by entering the median value of each quartile into the generalized linear model. Data are expressed as the mean (standard deviation) for normally distributed data, the median (interquartile range) for nonnormally distributed data, and the percentage (%) for categorical variables*.

### Serum Ionized Calcium Is a Prognostic Factor for Clinicopathology in EC Patients

Univariate logistic regression analysis reveals the relationship between LVSI and clinicopathologic relative features as age (OR = 1.4, 95% CI: 1.10, 1.8, *P* = 0.006), CA125 level (OR = 1.0, 95% CI: 1.0, 1.0, *P* < 0.001), menopause (OR = 1.7, 95% CI: 1.0, 2.8, *P* = 0.038), tumor grade (OR = 7.4, 95% CI: 4.5–12.2, *P* < 0.001), FIGO stage (OR = 2.9, 95% CI: 2.3, 3.8, *P* < 0.001), cervical invasion (OR = 8.0, 95% CI: 4.8–13.4, *P* < 0.001), myometrial invasion (OR = 11.4, 95% CI: 6.6–20.0, *P* < 0.001). And as for quartile of corrected serum calcium when normalized to Q1, Q3 (1.96–2.12 mmol/L) (OR = 2.7, 95% CI: 1.3–5.8, trend *P* = 0.009) and Q4 (>2.12 mmol/L) (OR = 3.3, 95% CI: 1.6, 6.9, trend *P* = 0.002) illustrates the elevation of corrected calcium level was a risk factor for clinicopathology in EC patients (Table 2).

**Table 2:** Univariate logistic regression analysis of in EC patients.

**Variables**	**Univariate analysis**	
	**OR (95%CI)**	***P*** **-value**
Age (y)	1.4 (1.1,1.8)	0.006
BMI (kg/m^2^)	0.9 (0.7,1.3)	0.705
Serum calcium (mmol/L)	2.4 (0.5,12.5)	0.287
Albumin (g/L)	1.0 (0.9,1.0)	0.173
CA125 (U/ml)	1.0 (1.0,1.0)	<0.001
Menopause	1.7 (1.0,2.8)	0.038
Tumor grade	7.4 (4.5,12.2)	<0.001
FIGO stage	2.9 (2.3,3.8)	<0.001
Cervical invasion	8.0 (4.8,13.4)	<0.001
Myometrial invasion	11.4 (6.6,20.0)	<0.001
**Corrected serum calcium (mmol/L)**		**P for trend**
Q1 ( ≤ 1.81)	1.0	
Q2 (1.82-1.95)	1.9 (0.8,4.1)	0.123
Q3 (1.96–2.12)	2.7 (1.3,5.8)	0.009
Q4 (>2.12)	3.3 (1.6,6.9)	0.002

*BMI, body mass index; OR, odds ratio; CI, confidence interval; FIGO, International Federation of Gynecology and Obstetrics; LVSI, lymph vascular space invasion*.

### The Influx of Extracellular Calcium Is the Origin of Estrogenic Effects on Calcium Homeostasis in Endometrial Cancer Cells

Maintaining the homeostasis both intracellularly and extracellularly of calcium concentrations is essential for cellular function. How elevated serum calcium affects the occurrence and development of endometrial cancer? Increasing evidence has suggested differences in regulating Ca^2+^ signaling between cancer cells and normal cells (12). In the previous study, With the effect of E2/E2-BSA, a significant increase in intracellular calcium in Ishikawa cells (ER-positive). To study whether E2-BSA-mediated changes in intracellular calcium levels in endometrial cancer cells originate from external calcium influx or internal calcium release and whether the increased extracellular calcium causes intracellular calcium rise. The intracellular concentrations of calcium ions in EC cells were detected by the Fluo-4 am calcium probe. E2-BSA can usually stimulate the level of intracellular calcium to increase; [Ca^2+^]cyt in Ishikawa and AN3CA (ER^+^) EC cell lines were distinctly enriched compared with that in HEC-1A and KLE cells (ER^−^) cells (Figure 1A).

**Figure 2:**
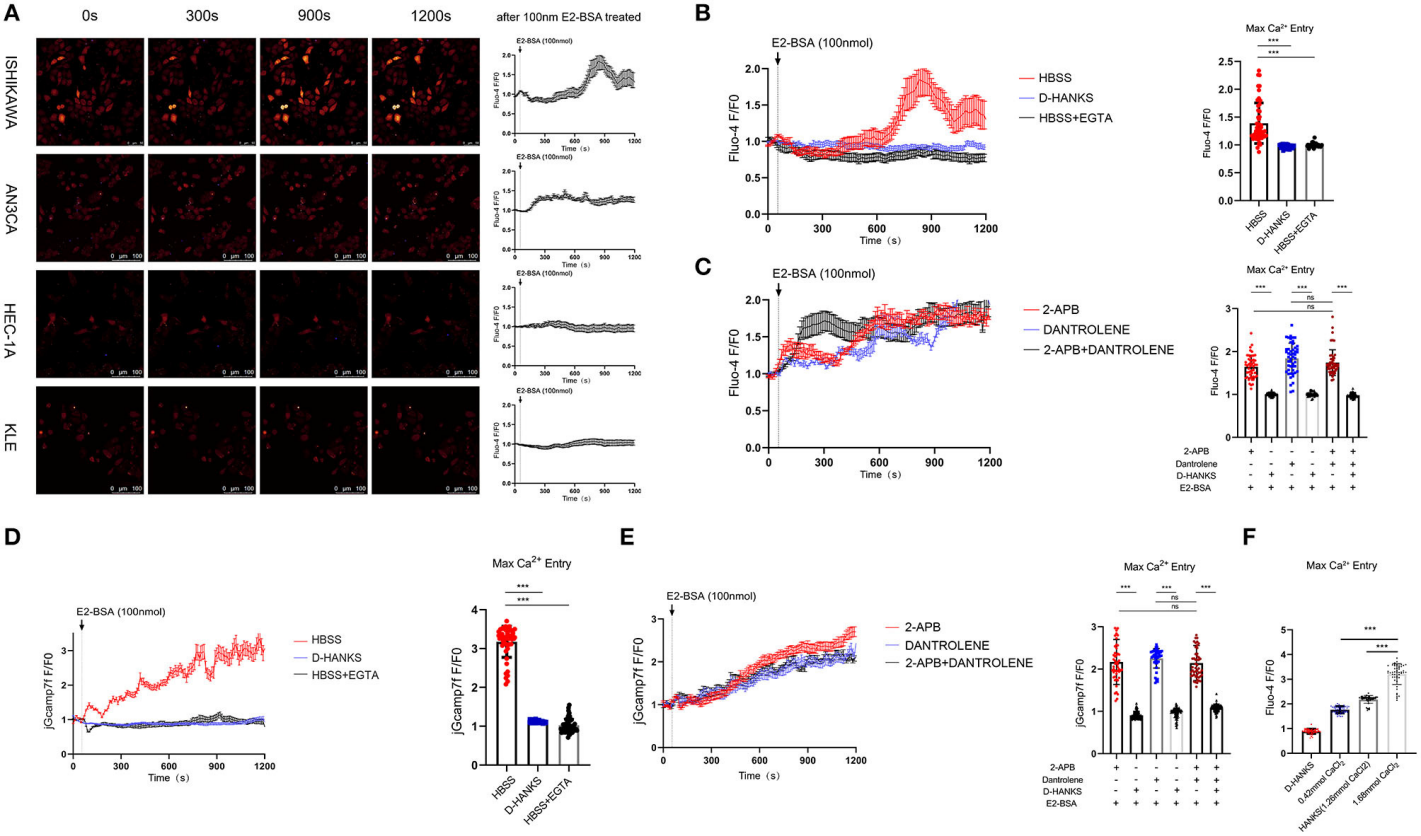
The calcium homeostasis regulated by estrogen in EC cells is derived from extracellular calcium influx but not the release of the endoplasmic reticulum. **(A)** The effect of E2-BSA of EC cell lines on calcium influx. Depicted are representative confocal images after adding 100 nm E2-BSA on the 60s. Average fluorescence intensity of each cell in the field (F), normalized to the non-specific background fluorescence (F0) to obtain the fluorescence intensity (F/F0). **(B)** E2-BSA stimulation of Ishikawa cells with calcium-free buffer solution D-HANKs or calcium chelating agent EGTA to remove extracellular calcium levels. *N* = 50. **(C)** E2-BSA stimulation of Ishikawa cells with inhibitors of endoplasmic reticulum calcium release (2-APB and Dantrolene 10μm) for 30 min. **(D,E)** GECI probe jGcamp7f transfected Ishikawa cells with E2-BSA stimulated calcium imaging. **(F)** Effect of different concentrations of calcium levels on estrogen-regulated calcium influx. All data are presented as the means ± SE. *n* = 50 for cell numbers and *n* = 3 for replicated. ns, non significant; ***p < 0.001. compared with the control group.

To identify the source of calcium changes, we monitored intracellular calcium alterations in the presence of Ca^2+^ in the normal HBSS buffer, in D-HANKS buffer (without Ca^2+^, Mg^2+^), and calcium chelating agent EGTA was used conditions. The results show that changes in intracellular calcium level caused by E2-BSA from the influx of extracellular calcium in the Ishikawa cell line, when Ca^2+^ is present outside the cell, and when the extracellular calcium is removed, the effect of E2-BSA is inhibited (Figure 2B, the *F*/*F*0 peak value has a significant difference). Inhibitors of endoplasmic reticulum calcium release (2-APB and Dantrolene) were used to block the maximum calcium storage endoplasmic reticulum at a single-cell level using Fluo-4 am imaging and jGcamp7f as a genetically encoded calcium indicator (GECI). When extracellular Ca^2+^ is present, the application of two endoplasmic reticulum calcium blockers, whether separately or combined, would not inhibit the elevation of [Ca^2+^]cyt induced by E2-BSA. The phenomenon is suppressed when extracellular calcium is removed (Figures 2C–E). The results showed that extracellular calcium is an important condition for E2-BSA to exert its effect by regulating the level of intracellular calcium. Calcium influx flow levels depend on different extracellular calcium concentrations during the same estrogen stimulation (Figure 2F). The changes in intracellular calcium levels were caused by the influx of extracellular calcium rather than the release of intracellular calcium storage. Further, we examined whether the estrogen-mediated calcium influx affects endometrial cancer cells' migration. Gap closure suggested that cells treated with E2-BSA combined CaCl_2_ had more motility in the Ishikawa cells (Supplementary Figure 1). Therefore, we concluded that E2-BSA induced calcium flux enhanced cell migration ability.

### The Influx of Extracellular Calcium May Be Involved in the Regulation of Autophagy and Mitochondrial-Related Pathways

**Figure 3:**
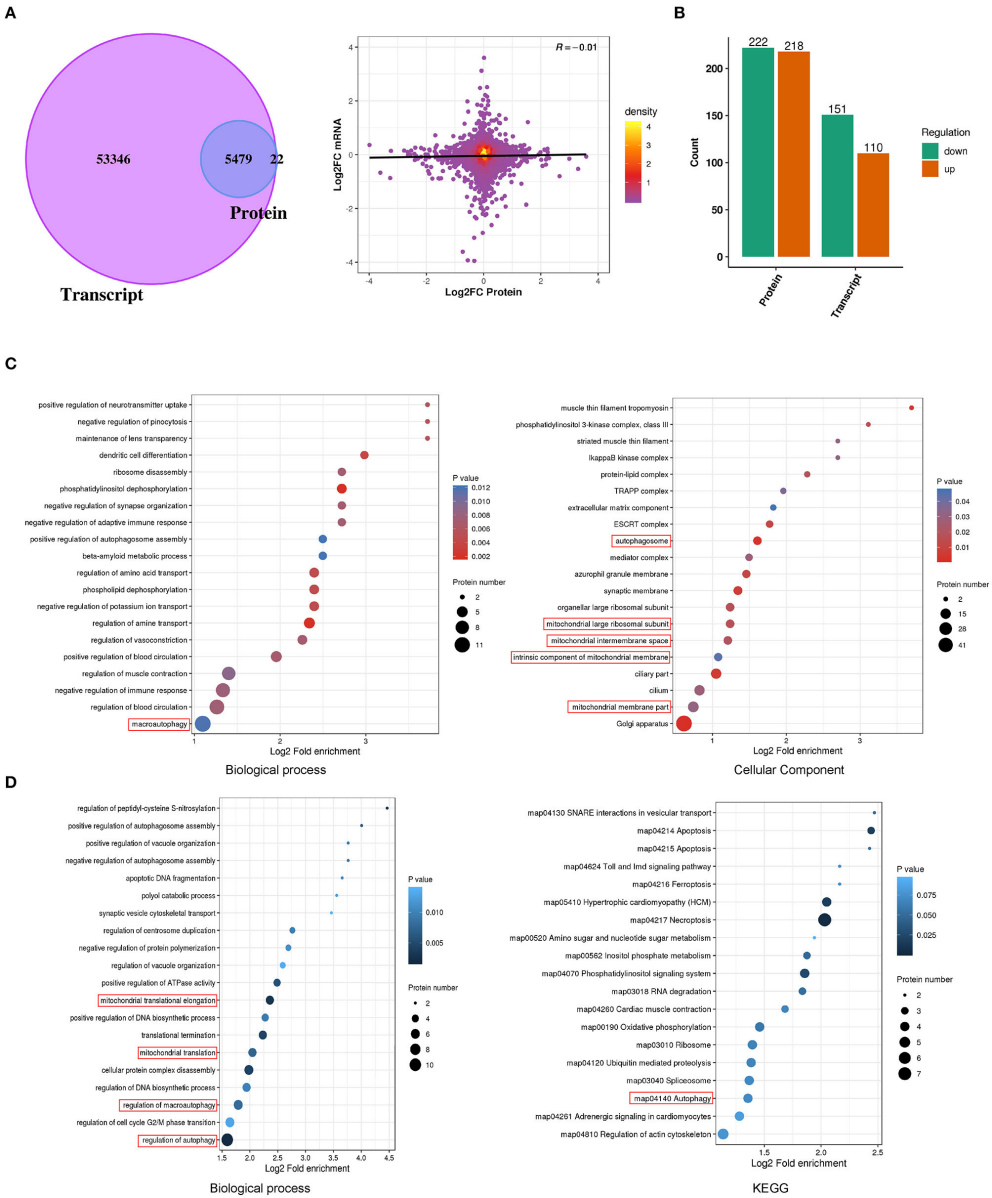
Proteomic combined with transcriptomic analysis of E2-BSA treated Ishikawa cell line. **(A)** Venn diagram and volcano chart depicting the overlap of proteomic and transcriptomic relative genes. **(B)** The distribution of genes up- and down-regulated in protein and transcript, respectively. **(C)** Results of GO and KEGG analysis of the up-regulated genes in proteomic. **(D)** Results of up-regulated proteomic combined with unchanged transcriptomic genes of GO and KEGG analysis.

The mechanisms of E2-BSA carcinogenesis and EC progression are only partially understood. Transcriptomic and proteomic analyses on Ishikawa cells treated with E2-BSA were performed to learn more about the processes. After the 6 h of E2-BSA administration, we quantified 58825 mRNAs and 5519 proteins in total, providing a multidimensional atlas (Figures 3A,B). The atlas reveals a proteogenomic state of E2-BSA treated cells. 5479 proteins (99.3 %) were uniquely mapped to overlapping mRNAs using integrative functional genomic analyses. The number of total and differentially expressed genes (DEGs) showed either upregulation or downregulation at *P*-value < 0.05 in both E2-BSA treated group and NC group. The distribution of the number of differentially expressed genes and proteins is shown in Figure 3C.

A total of 222 down-regulated genes (Log2FC <1.5) and 218 up-regulated genes (Log2FC > 1.5) were identified in the protein profile; 151 down-regulated genes and 110 up-regulated genes were identified in the transcriptome (Figure 3B). Gene ontology (GO)enrichment was carried out for the biological analysis of DEGs, and the information of their putative functions was predicted in terms of molecular functions (MFs), biological processes (BPs), and cellular components (CCs). In order to target the pathways that the DEGs were involved in, the Kyoto Encyclopedia of Genes and Genomes (KEGG) analysis was carried out. We performed combined transcriptome and proteome analyses. As a result, differential enrichment is involved in the regulation of autophagy, cytosol, protein assembly mitochondrion, and regulation of autophagy pathway (map04140), actin cytoskeleton (map04810) in the KEGG pathway (Figures 3C,D). These results indicate that the estrogen-mediated increased intracellular calcium may be involved in regulating downstream autophagy and mitochondrial-related pathways.

### Removing the Extracellular Calcium Inhibit Mitochondrial Ca^2+^ Overload and Prevent the Increase of Mitochondrial ROS

**Figure 4:**
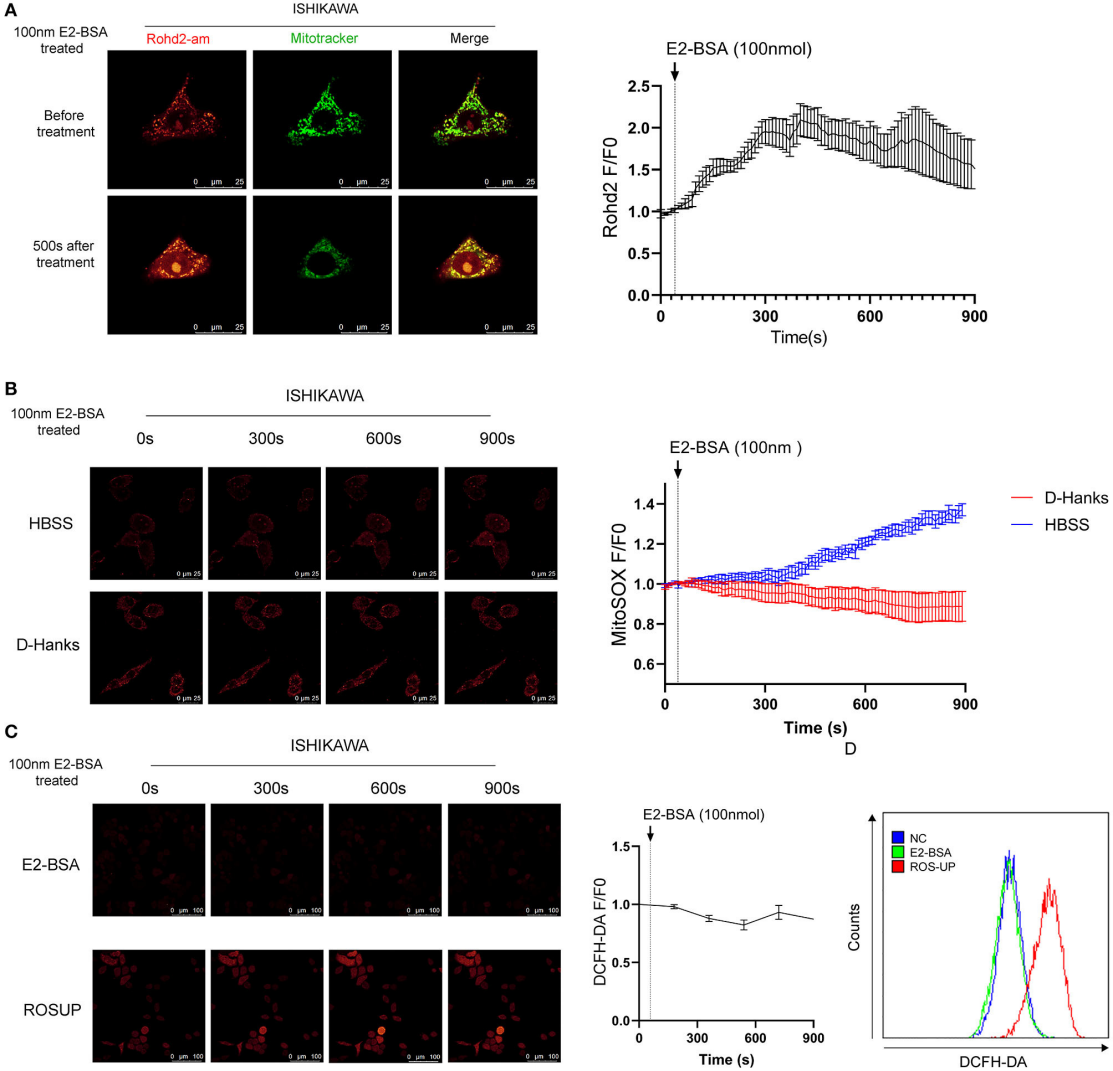
The influence of estrogen-regulated calcium influx in the mitochondrion of Ishikawa cell line. **(A)** The effect of E2-BSA induced calcium influx of mitochondrion calcium in the Ishikawa cell line. Rohd2-am (red) was used to label the calcium of mitochondrion. **(B)** Influence of calcium influx of mitochondrion ROS. Magnification 63 ×. Scale bar 25μm. **(C,D)** Confocal images and flow cytometry of the influence of calcium influx of intracellular total ROS level detected by DC-FHDA. Magnification 63 ×. Scale bar 100μm. All data are presented as the means ± SE. *n* = 50 for cell numbers and *n* = 3 for replicated.

Autophagy is the process by which cellular material is delivered to lysosomes for degradation and recycling. Several studies have underlined the importance of Calcium ions (Ca^2+^) signaling for autophagy. Intracellular Ca^2+^ is shuffled between the cytoplasm and the major Ca^2+^ stores, the endoplasmic reticulum (ER), the mitochondria, and the lysosomes to trigger signaling cascades (13). We wondered whether the Ca^2+^ worked in E2-BSA-mediated in the mitochondrion ([Ca^2+^]mito) of endometrial cancer cells to cause autophagy. We visualized mitochondrial Ca^2+^ with Ca^2+^ indicator Rhod-2 specifically in mitochondria. The results showed that E2-BSA increased the intensity of Rhod-2 fluorescence in mitochondria, and the peak appeared at about 350–550 s, indicating that it promoted mitochondrial Ca^2+^ uptake, consistent with E2-BSA causing the increased intracellular calcium level (Figure 4A).

The increased mitochondrial ROS can promote tumorigenesis, cancer progression, metastasis. The autophagy-targeted degradation of mitochondria is accomplished by a selective form of autophagy, named mitophagy. Mitophagy can be initiated mitochondrial damage caused by hypoxia, chemical uncouplers, or ROS (14). We further explore whether the regulation of elevated calcium signals can cause changes in the active oxide ROS of endometrial cancer cells. DCFH-DA was used to label the total intracellular ROS level, and Mito-SOX was used to label the mitochondrial ROS. Under a confocal microscope, the ROS level and mitochondrial ROS level of Ishikawa cells were dynamically observed under the effect of E2-BSA for 900 s. Mitochondrial Ca^2+^ overload can lead to the increase of mitochondrial ROS.

In contrast, the fluorescence intensity of DCFH-DA did increase; the flow cytometry results are consistent with the confocal experiment, as shown in Figure 4B. To test if removing the extracellular calcium inhibits mitochondrial Ca^2+^ overload, we treated Ishikawa cells with 100 nm E2-BSA in HBSS/D-Hanks buffer. Mitochondrial Ca^2+^ overload was absent in deficient extracellular calcium, indicating that mitochondrial Ca^2+^ overload and its downstream activators were required for extracellular calcium (Figures 4C,D).

### Inhibition of Extracellular Calcium Influx Induces Lysosomal Dysfunction

**Figure 5:**
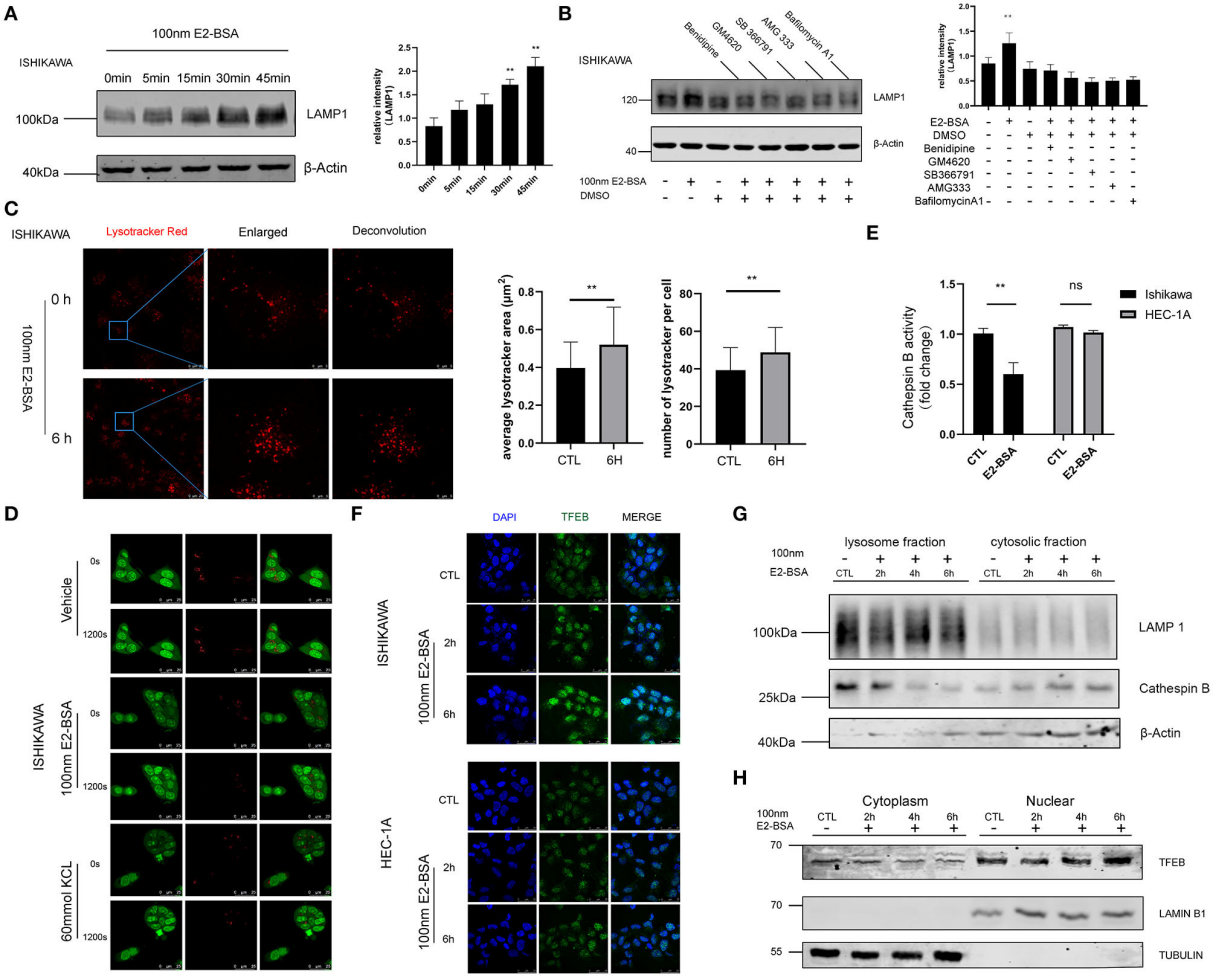
The influence of estrogen-regulated calcium influx in Ishikawa and Hec-1A cell line lysosome. **(A)** An E2-BSA significantly promoted the LAMP1 expression in Ishikawa cells with rapid effect. **(B)** Inhibition of extracellular calcium influx reduced lysosomal response. **(C)** Effect of E2-BSA on lysosomes. The lysosomes were labeled by 5 nM Lyso-Tracker Red (LTR) and examined confocal. Magnification 63 ×. Scale bar 25 or 5μm. **(D)** Ishikawa cells stained with 5 μg/ml acridine orange (AO) for 15 min were then treated with E2-BSA and imaged under a confocal microscope (scale bar: 25 μm). **(E)** E2-BSA treatment causes differential regulation in Ishikawa or HEC-1A cells. The activities of CTSB were measured by the fluorometric method using commercially available kits. All data are presented as the means ± SE. *n* = 3–4. **(F,G)** E2-BSA stimulated external calcium influx can activate the nuclear translocation of TFEB. **(H)** Effect of TFEB nuclear shift after E2-BSA treatment. All data are presented as the means ± SE. *n* = 50 for cell numbers and *n* = 3 for replicated. ns, non significant; **p < 0.01 compared with the control group.

The lysosome is an acidic, membrane-bound organelle containing hydrolytic enzymes such as cathepsins, which are the main executioners of protein degradation in autophagy. Cells degrade damaged or permeabilized lysosomes through lysophagy (14). To test if elevated Ca^2+^ was linked to lysosomes in endometrial cancer cells, the confocal microscope was used to explore the changes of morphology and number of lysosomes labeled with Lysotracker and processed the image with Huygens deconvolution. The results demonstrated an increase in the number of lysosomes. They expressed the lysosomal marker LAMP-1 after being treated with 100 nm E2-BSA for 6 h compared with the control group, which may be related to the effect of calcium flux mediated by estrogen (Figures 5A,C). We wondered if reducing Ca^2+^ uptake would prevent lysosomes activity. Benidipine, GM4620, SB 366791, AMG333 represent different classes of drugs that inhibit Ca^2+^ channels and found that they did, which is similar to the inhibition of lysosomal effects of Bafilomycin, suggesting that inhibition of extracellular calcium influx induce a lysosomal dysfunction (Figure 5B). E2-BSA can increase lysosomal membrane permeabilization (LMP) by causing external calcium influx under short-term action and regulating lysosomal function (Figure 5D). Lysosomes with increased permeability are often accompanied by leakage of lysosomal enzymes and changes in activity (Figure 5E). We finally aimed to test whether also E2-BSA- mediated Ca^2+^ controls lysosomal activity in endometrial cancer cells, the result was verified by Western blot, Cathepsin B (CTSB) was expressed in the lysosomal fraction of Ishikawa cells, E2-BSA treatment caused the CTSB to enter the cytoplasm (Figure 5G). Accordingly, the increased Ca^2+^ caused by E2-BSA is an essential contributor to lysosomal activity.

### The Influx of Extracellular Calcium Regulated Autophagy by TFEB Nuclear Translocation

Disrupting organelle homeostasis via calcium flux inhibition may have unintended consequences. How organelle-specific autophagy pathways intersect with other organelle functions is also unclear. The role of nuclear transcription factor EB (TFEB) is mainly to regulate lysosomal biogenesis and autophagy (15). Whether estrogen up-regulates, intracellular calcium levels are also related to nuclear effects of gene transcription needs to explore further. The application of immunofluorescence and Western blot experiments further confirmed that: in the Ishikawa cells, E2-BSA treatment for 6 h was followed by the relocation of TFEB from the cytoplasm to the nucleus. While the TFEB are scattered located in the cytoplasm in the control group. The results showed that E2-BSA stimulated external calcium influx can activate the nuclear translocation of TFEB, as shown in Figures 5F,H. In sum, estrogen-mediated extracellular calcium influx may be the main regulatory factor related to autophagy regulated by TFEB and plays a vital role in linking the nongenomic and genomic effects in endometrial cancer.

## Discussion

The worldwide incidence of endometrial carcinoma (EC) is increasing, and the death rate has accelerated from 0.3% per year from 1997 through 2008 to 1.9% per year from 2008 through 2018 (1). Exposure to endogenous and exogenous estrogens is the primary risk factor for the development of EC. EC had historically been categorized as 2 pathogenic types, although it lacks sufficient discriminative ability to categorize tumors or to guide the treatment of EC today (2). The mechanism of estrogen works involves different physiological responses, and we evaluated its effect on EC growth and estrogen non-genomic signaling and genomic signaling. The non-genomic effect does not involve specific receptors in the nucleus but activates related signal pathways in the membrane/cytoplasmic, such as pathways mediated by membrane estrogen receptor (mER) or G protein-coupled receptors, ion channels, and other receptors, and causing an increase in intracellular Ca^2+^ concentration (7, 16).

The role of Ca^2+^ in malignant transformation, tumor progression, and response to treatment has been considerably re-evaluated over the past decade (17). Still, few studies have evaluated the importance of calcium levels as a blood-based biomarker. In this study, we analyzed the differences in serum calcium to identify links between serum calcium and the clinicopathological characteristics of endometrial cancer to evaluate the prognostic value of serum calcium levels in endometrial cancer patients. To our knowledge, this is the first study indicating the prognostic value of circulating levels of calcium in endometrial cancer. We found an overall association between increasing ionized calcium and menopausal status, age, FIGO staging, lymph node metastasis, and LVSI myometrial invasion through the retrospective analysis of clinical data of endometrial cancer patients cervical invasion. Albumin-corrected serum calcium is more accurate in measuring calcium status. In our study, the increased albumin-corrected serum calcium is a risk factor for LVSI in the Q4 (>p75) partition in the EC patients subgroup. We found that elevated serum calcium associates significantly with lymph node metastasis, LVSI, and deep myometrial infiltration might point to a potential role for the serum calcium in selecting patients for lymphadenectomy or adjuvant treatment. Combined with CA125, identification of serum calcium as a new cancer biomarker in blood samples which could provide additional information, is attractive.

Some studies have reported that serum calcium has been identified as a prognostic indicator for many human malignancies, such as prostate cancer and nasopharyngeal carcinoma (18, 19). Calcium ions are the most common second messengers that play an essential role in cancer development by widely regulating diverse cellular functions. The concentration of free Ca^2+^ in the cytosol ([Ca^2+^]c) has to be maintained at the nanomolar range. Both Ca2+ entries can elicit increases in [Ca2+]c from the extracellular space through plasma membrane (PM) channels and Ca^2+^ release from intracellular stores (20). However, studies regarding the high serum calcium levels are involved in the pathogenesis of endometrial cancer have not yet been conducted. Non-genomic effects of estrogen involve activation of signal-transduction mechanisms with the subsequent changes of intracellular calcium has been shown by our group in endometrial cancer (7). In order to exclude the influence of genomic events mediated by the nuclear estrogen receptors (nERs), researchers utilize membrane-impermeable conjugates of E2, steroid-BSA conjugates were initially synthesized for use as immunogens, such as commercial used E2 conjugated bovine serum albumin (E2-BSA), were first carried out in the 1980s (21, 22). In contrast to E2, the non-impermeable E2-BSA only binds to membrane ER receptors, which induced rapid non-genomic effects. Our previous study demonstrated that G protein-coupled estrogen receptor (GPER) as the non-nuclear ER in the plasma membrane is activated by E2-BSA and leads to calcium mobilization for estrogen action (7).

We further hypothesize that the changes in intracellular calcium levels originate from the influx of extracellular calcium. The influx of calcium levels affects the calcium homeostasis of organelles and organelle functions, ultimately interacts with gene transcription effects, and regulates the occurrence and progression of endometrial cancer. To confirm this, we monitored intracellular calcium alterations in the presence of estrogen in endometrial cancer cell lines. The results showed that the changes in intracellular calcium levels caused by E2-BSA were caused by the influx of extracellular calcium rather than the release of intracellular calcium. And when the extracellular calcium is removed, it can no longer cause the up-regulation of intracellular calcium levels.

Estrogen plays an essential role in the process of extracellular calcium influx, and it is tightly controlled by complex mechanisms involving either genomic effects or non-genomic effects. In this study, we combined proteomics and transcriptome analysis of the downstream pathway changes after E2-BSA-mediated calcium influx, high-throughput sequencing (RNA-seq) was performed to assess the transcriptomic changes and the key genes involved in response to estrogen, functionally annotated with gene ontology (GO) terms and complex regulatory pathways (KEGG), which were mainly associated with autophagy and mitochondrial-related pathways. Autophagy can promote and inhibit tumor growth in different contexts (23, 24), the mechanisms are complicated, and organelle-specific autophagy pathways may be involved. Mitochondria are the powerhouse of the cells and participate in many regulatory functions, including Ca^2+^ homeostasis. The moderate Ca^2+^ signal can promote the increase of ROS in tumor cells and then activate the cancer-promoting signal transduction, enhance cell survival and proliferation, mediate DNA damage and genetic instability and lead to further mutations. Dysfunctional mitochondria and increased mitochondrial ROS can promote tumorigenesis, cancer progression, metastasis, and drug resistance (14). Lysosomes are related to the degradation of macromolecules and play an essential role in cell secretion, signal transduction, and energy metabolism. As the main executioners of protein degradation, Lysosomes, and intracellular calcium reservoirs outside the endoplasmic reticulum, play a vital role in the process of autophagy and are closely related to cancer cell metastasis. The aggressiveness of cancer cells relies heavily on effective lysosomal functions to provide the energy needed for their rapid division and migration to other organs. Cancer cells alter their lysosomal compartment, including size, localization, cathepsin expression, and activity. Its membrane stability and permeability are critical to autophagy lysosomes (14, 25). Therefore, our dissection of the pathway reveals interconnections between Ca^2+^ that span different organelles focus on the effect of increased intracellular Ca^2+^ on the mitochondria and lysosomes. The results indicated that E2-BSA promoted mitochondrial Ca^2+^ uptake, mitochondrial Ca^2+^ overload can lead to the increase of mitochondrial ROS. E2-BSA in EC cells can also increase the number of lysosomes and expression of the LAMP-1, changing the lysosomal membrane permeabilization (LMP) accompanied with leakage of Cathepsin B. We tested if removing extracellular calcium would remove mitochondrial Ca^2+^ overload, different classes of calcium channel blockers shown to inhibit lysosomal dysfunction. These results confirmed that extracellular calcium is required for cytosolic Ca^2+^ homeostasis and estrogen-induced autophagy pathway. Whether Ca^2+^ has different roles in the inter-organellar relationship in endometrial cancer pathogenesis remains determined. What is clear is that E2-BSA-treated EC cells undergo a specific form of regulation involving multiple organelles associated with autophagy.

Autophagy is regulated by lysosomal proteinases, cathepsins B (26), a fundamental biological function of cathepsin B in providing a checkpoint for homeostatic maintenance of the lysosome population and basic recycling functions in the cell. Cathepsin B appears to play an essential role in tumorigenesis and the progression of EC (27, 28). In this study, estrogen-mediated extracellular calcium influx can lead to the release of lysosomal CTSB, and the decreased CTSB activity is related to the nucleus and activation of TFEB. In supporting cellular homeostasis, not only do lysosomal signals actively influence nuclear transcription by altering lysosomal positioning and promoting nuclear translocation of chaperone proteins and TFs, but nuclear transcription also dynamically regulates genes involved in lysosomal biogenesis and functions and autophagy. Several TFs are implicated in this nuclear control of lysosomal adaptation to ensure that lysosomes can adjust their values and activities in the context of environmental fluctuation (29). And further studies should be conducted to explore the crosstalk between the non-genomic effect and the genomic effect of estrogen in EC linked by TFEB.

In conclusion, our retrospective study convincingly demonstrated serum calcium is a risk factor for clinicopathology in the Q4 (>p75) partition in the EC patients' subgroup, and serum calcium levels can act as a prognostic indicator. The elevated intracellular calcium-mediated by non-genomic effects of estrogen originates from the influx of extracellular calcium. The influx of calcium levels affects the calcium homeostasis and functions of mitochondria and lysosomes, ultimately regulating autophagy pathways by TFEB involved in the occurrence and progression of endometrial cancer. Our findings revealed that serum calcium level was significantly related to poor outcomes. The extracellular calcium influx induced by estrogen should be targeted to original preventive strategies in EC. The mechanisms of malignancy-associated serum calcium level in organelle calcium homeostasis should be identified.

## Data Availability Statement

The datasets presented in this study can be found in online repositories. The names of the repository/repositories and accession number (s) can be found in the article/Supplementary Material.

## Ethics Statement

Written informed consent was obtained from the individual(s) for the publication of any potentially identifiable images or data included in this article.

## Author Contributions

BS, JH, JianW, JZ: conception and design. BS, JH: collection and assembly of data. BS, JH, JZ: manuscript writing. All authors: data analysis and interpretation and final approval of manuscript.

## Funding

This work was supported by grants from the National Natural Science Foundation of China (No. 81902635, 82072861, and 81874108) and the Fujian Provincial Natural Science Foundation of China (No. 2020J05276).

## Conflict of Interest

The authors declare that the research was conducted in the absence of any commercial or financial relationships that could be construed as a potential conflict of interest.

## Publisher's Note

All claims expressed in this article are solely those of the authors and do not necessarily represent those of their affiliated organizations, or those of the publisher, the editors and the reviewers. Any product that may be evaluated in this article, or claim that may be made by its manufacturer, is not guaranteed or endorsed by the publisher.

## Supplementary Material

Supplementary Figure 1 E2-BSA induced calcium influx promoted cell migration in Ishikawa cells. Wound-healing assay for Ishikawa cells after E2-BSA/CaCl_2_/E2-BSA+ CaCl_2_ treatment. Scale bar = 200 μm.
