# Supplementary figures and images for: Estrogen-Induced Extracellular Calcium Influx Promotes Endometrial Cancer Progress by Regulating Lysosomal Activity and Mitochondrial ROS
Source: Front Med (Lausanne). 2022 Feb 10;9:835700. doi: 10.3389/fmed.2022.835700 (PMC8866192; doi:10.3389/fmed.2022.835700)

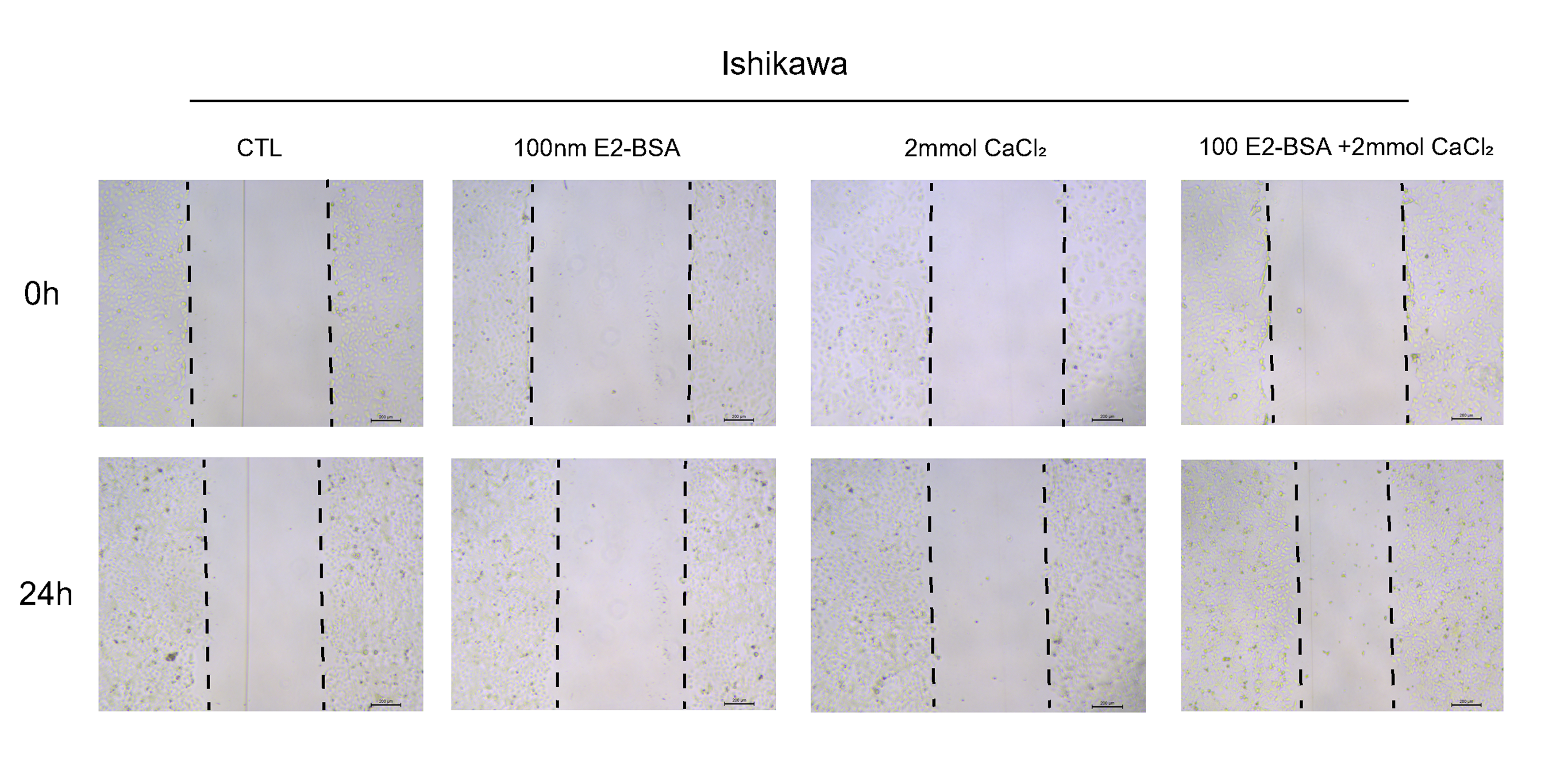

Supplement: Supplementary Figure 1 — E2-BSA induced calcium influx promoted cell migration in Ishikawa cells. Wound-healing assay for Ishikawa cells after E2-BSA/CaCl2/E2-BSA+ CaCl2 treatment. Scale bar = 200 μm. [file Image_1.TIFF]
